# Potential role of dental pulp stem cells conditioned medium for odontoblastic differentiation

**DOI:** 10.1186/s40659-022-00380-8

**Published:** 2022-03-04

**Authors:** Benson Koh, Farynna Hana Ab Rahman, Najwa Amira Matlan, Manissha Rajan, Aimi Yasmin Musta’ain, Mohamad Ridhwan Mohd Jeffry Lee, Roszalina Ramli, Siti Salmiah Mohd Yunus, Ruszymah Binti Hj Idrus, Muhammad Dain Yazid

**Affiliations:** 1 Centre for Tissue Engineering and Regenerative Medicine, Faculty of Medicine, Universiti Kebangsaan Malaysia Medical Centre, Jalan Yaacob Latif, Cheras, 56000 Kuala Lumpur, Malaysia; 2 Department of Oral and Maxillofacial Surgery, Faculty of Dentistry, Universiti Kebangsaan Malaysia, Jalan Raja Muda Abdul Aziz, 50300 Kuala Lumpur, Malaysia

**Keywords:** Odontoblast, Wharton’s Jelly mesenchymal stem cells, Dental pulp stem cells, Conditioned medium, Dentures

## Abstract

**Background:**

Functional bioengineered tooth regeneration using autologous or allogeneic alternative differentiated cells sources are thought to have a great potential in replacing conventional dentures. This study investigated the potential of dental pulp stem cells (DPSCs) conditioned medium for odontoblastic differentiation of Wharton’s jelly mesenchymal stem cells (WJMSCs). The DPSCs derived from healthy adult permanent first molars were cultured at high confluence prior to conditioned medium collection. The WJMSCs were cultured in six different treatments, with varying ratios of culture media to DPSCs-conditioned medium. MTT assay was used to measure the rate of proliferation of WJMSCs, while immunocytochemistry staining was utilised to detect the expression of dental matrix protein 1 (DMP-1). The deposited calcium was detected and analysed via Alizarin-Red Staining (ARS).

**Results:**

It was found that the proliferation of WJMSCs cultured under the mixture of complete medium and DPSCs conditioned medium showed significantly lower than the control; presumably the cells started to exit proliferative state prior differentiation. In 14 days of induction, the cells in all treatments showed osteoblastic-like morphology, calcium compound deposits were observed at day 7, 10 and 14 of differentiation suggested that DPSCs conditioned medium could lead to osteoblastic/odontoblastic differentiation. However, the DMP-1 protein can be seen only expressed minimally at day 14 of conditioned medium induction.

**Conclusions:**

In conclusion, DPSCs conditioned medium appeared as a potential odontoblastic induction approach for WJMSCs. To further investigate the stimulatory effects by DPSCs conditioned medium, specific signalling pathway need to be elucidated to enhance the differentiation efficiency.

## Introduction

Dentures, or artificial teeth, have been dated back to 700 BC in northern Italy where human or animal teeth were used [1]. It is now more common to use dentures made from resin or porcelain. Nonetheless, complications arise such as allergy to polymethylmethacrylate (PMMA), denture stomatitis, denture irritation, ulceration, fracture of denture base and even loss of artificial teeth [2]. Poor hygiene of dentures may cause an infection and eventually worsen the condition by irreversibly damaging the permanent teeth. Endodontic approach that has been widely used currently to treat damaged teeth is pulp amputation followed by apical closure induction using synthetic biocompatible material at the root apex or better known as root canal treatment [3]. It is unfortunately unable to stimulate or induce pulp regeneration. The intrinsic pulp sensation also will not be recovered from a root canal treatment and may even result in an inability to detect secondary infections hence, dental pulp is crucial in ensuring continuity of root redevelopment after a tooth damage.

In the advancement of technology, tooth implantation is considered as an effective therapy for tooth avulsion due to trauma caused by a sports injury or motor vehicle accident. Alas, the disadvantage of using this approach is, it is highly dependent on the vitality of the periodontal ligament [4]. It is a race against time as the cells remained at the periodontal ligament have a short lifespan of 2 h if not preserved in an appropriate medium if an avulsed tooth functions were to be recovered. Dental implants or dentures despite being used in clinical practice by dentists it can fail and will not adapt to surrounding bones. In comparison of dental implants and tooth regeneration, tooth regeneration is the best treatment modality because regenerated periodontal bone remodels with existing alveolar bones, it stimulates bone regeneration along with tooth regeneration and regenerated tooth has native defence in its dental pulp and periodontal tissue [5, 6].

Stem cells, in particular, have attracted much attention. There have been multiple sources of MSCs being used in recent odontoblastic differentiation experiments worldwide, for instance some are using Human Skeletal Muscles Stem Cells, Bone Marrow MSCs and Dental Pulp Stem cells (DPSC) [7, 8]. However, Wharton Jelly Mesenchymal Stem cells (WJMSCs) are a more readily accessible source of stem cells as compared to bone marrow MSCs and instead of throwing away and wasting potentially good multipotent stem cells they are best be used in regenerative engineering [9, 10]. DPSC is used as a conditioned medium for differentiation of WJMSCs because DPSCs has the best environment for odontogenesis and also for its ability to secrete specific factors for new nerve innervation [11].

Odontoblasts are gaining interest in the field of dental regeneration and repair as a promising future treatment modality that could increase the longevity of pulp less teeth [12]. This is because odontoblasts demonstrate the ability to form dentin-pulp complex. The dentin-pulp complex is the key in maintaining overall structure and integrity of the tooth [13, 14]. Odontoblast that resides in the pulp has the property to secrete extracellular materials and proteins that are responsible for tooth mineralization in order to form dentin.

Therefore, as usage of dentures and bridges comes with complications and odontoblast has been found to be useful in tooth regeneration. A study of differentiation of WJMSCs into odontoblast-like cells using DPSC-conditioned medium is a promising possibility for tooth regeneration.

## Results

### Cells isolation and culture and conditioned medium production

**Fig. 1:**
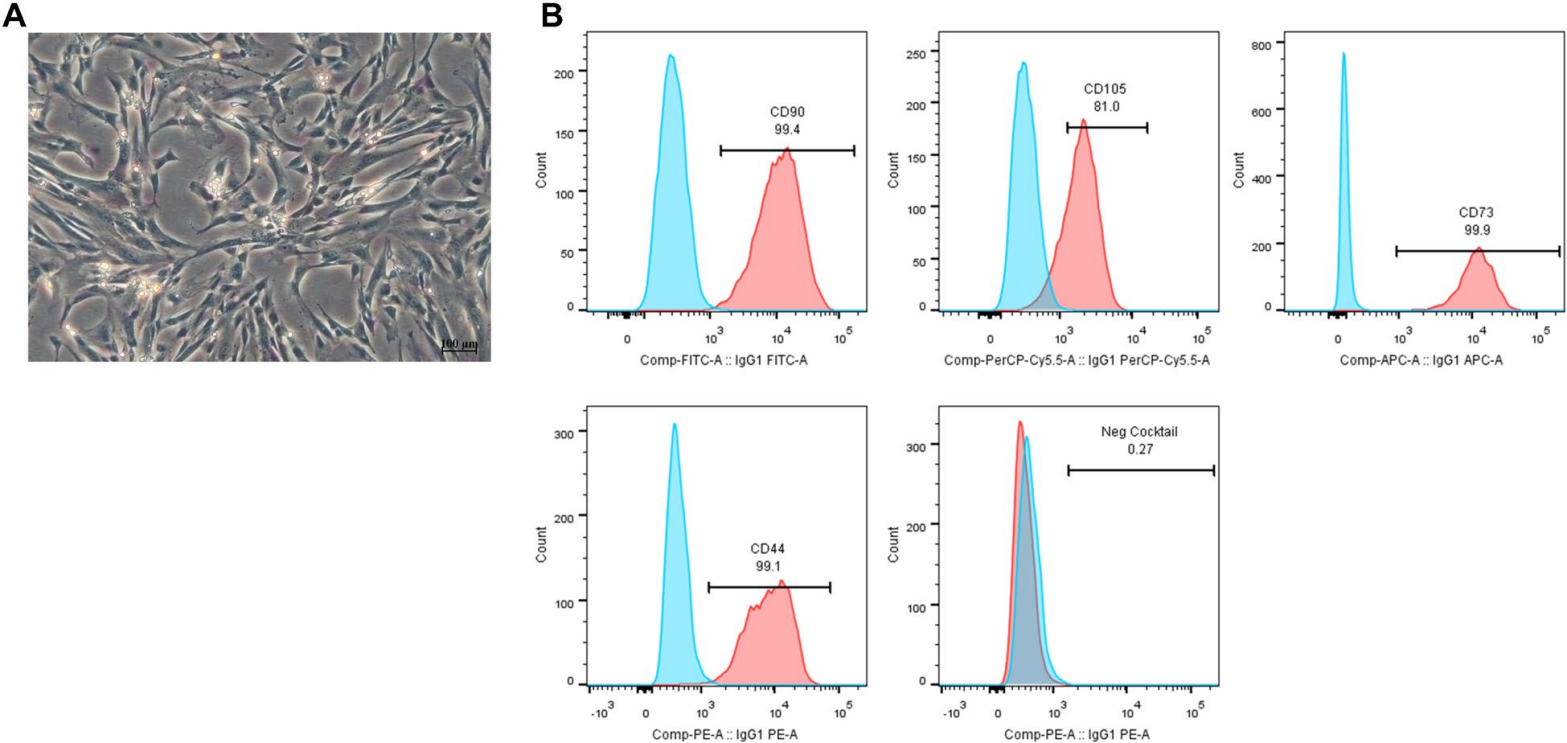
Morphology and immunophenotypic characteristic of isolated Wharton's Jelly MSCs. **A** Fibroblastic morphology of the isolated WJMSCs. **B** The expression of MSC positive (CD90, CD105, CD73, CD44) and negative (CD34, CD11b, CD19, CD45, HLA-DR in cocktail) markers. The light blue represents the respective isotype control, while the red represents WJMSCs

Wharton’s jelly mesenchymal stem cells (WJMSCs) were isolated from the Wharton’s jelly of umbilical cords. After 3–4 days in culture, it was clearly observed that the cells, principally, exhibited a fibroblast-like morphology (Fig. 1A). The tissue masses that failed to attach to the culture flask were removed, and the conditioned medium was replaced every 3 to 4 days. The cells gradually took on a typical fibroblast-shape after 7-day culture period and arranged themselves in parallel lines after 10 days. By that time, they had reached 80% confluency for passaging. Once passaged, the cells expanded rapidly and were passaged approximately once every week. The MSCs expanded were expressed positively in CD90, CD105, CD73, CD44 and lack expression of negative markers CD34, CD11b, CD19, CD45, HLA-DR (Fig. 1B).

Dental pulp stem cells (DPSCs) were successfully isolated from stem cells from human exfoliated deciduous teeth (SHED), according to Zainal Ariffin et al. [15]. These cells were positive for DPSCs-specific markers and exhibited typical fibroblast-like morphology. After 4 passages and approximately 70–80% confluent, the isolated DPSCs were cultured again to get the DPSCs-conditioned medium (DPSCs-CM) which were used for the odontoblast induction.

### Examination of the effect of DPSCs-CM on WJMSCs via MTT assay

**Fig. 2:**
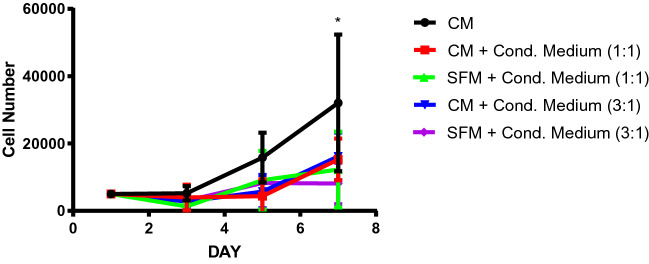
Effect of DPSCs Conditioned Medium on WJMSC viability. *CM* complete media, *Cond. Medium* conditioned media, *SFM* serum free media. All graphs represent an average of three repeats from different samples. All data are presented as mean ± S.D and were statistically analyzed using the student t-test (Microsoft Excel) and one-way ANOVA. Significantly different: *(p < 0.05) and **(p < 0.01) as compared to Day 1 samples

The WJMSCs were then analysed after 1, 3, 5, and 7 days to examine the effect of DPSCs conditioned medium on the growth of WJMSCs. This was done by measuring the proliferation rate of WJMSCs via MTT assay (Fig. 2). Based on the results obtained, there was no significant difference between the WJMSCs seeded in the 100% complete media than that of the cells treated with the DPSCs-CM and secretome with different ratios. However, significant increases (p < 0.05) of cell number observed on WJMSCs cultured in group Complete DMEM:CM 1:1, Complete DMEM:CM 3:1, and Serum-free DMEM:CM 1:1 from day 1 to day 7, indicating that the cell proliferation does not inhibit.

### Odontoblasts induction

After 4 passages, the isolated WJMSCs were prepared for odontoblasts induction. The WJMSCs were cultured in 6 different treatments. The positive control in this study was the WJMSCs cultured in StemPro osteogenesis differentiation kit, while the negative control was the flask with the cells cultured in 100% complete medium.

**Fig. 3:**
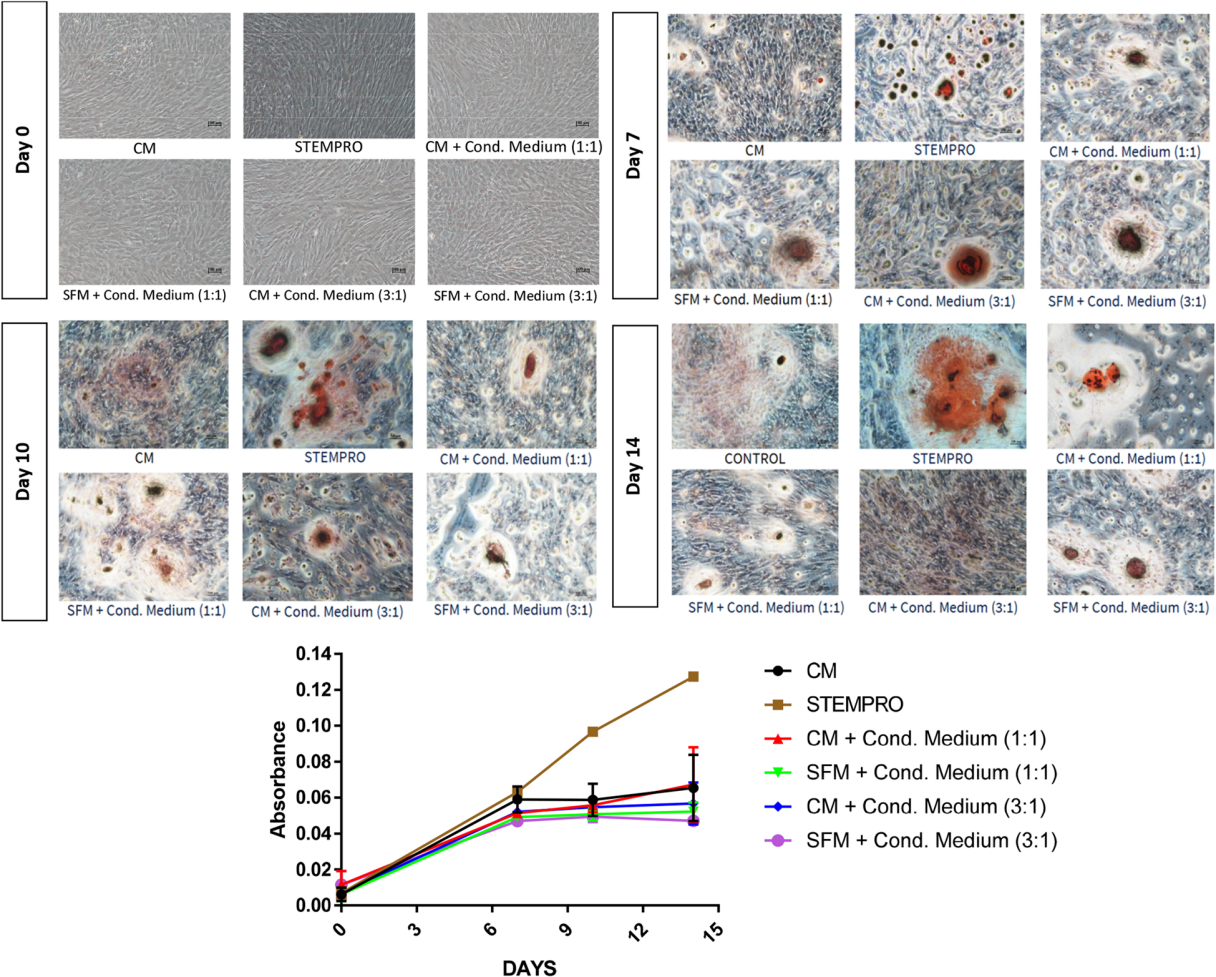
ARS observed under light microscope at Day 7, 10 and 14, showing calcium deposition; magnification =  × 40. Quantification of ARS; Absorbance measured at OD = 450 nm. *CM* complete media, *Cond. Medium* conditioned media, SFM, serum free media

The visualisation of the deposition of calcium in the differentiated cells, which indicates the presence of ongoing osteogenesis, was aided by Alizarin Red staining (ARS) (Fig. 3), done after 7, 10 and 14 days of induction. Microscopically, the differentiated cells treated in the StemPro and the DPSCs-CM were observed to form mineralized nodules in the 14-day culture. However, the mineralization observed in the differentiated cells in the DPSCs-CM treatments was not as obvious as that observed in the positive control (StemPro). As expected, less mineralization was noted in the differentiated cells in the negative control group at the end of the 14-day culture.

Similarly, the results from the quantitative analysis of ARS shows that only the differentiated cells in the positive control exhibited significantly higher ARS concentration evidenced by increased absorbance by the end of the 14-day culture. This result indicated that there was no significant difference between the control media and the DPSCs-CM in stimulating the differentiated cells mineralization potential.

### Determination of DMP1 protein expression on differentiated cells from WJMSCs via immunocytochemistry

**Fig. 4:**
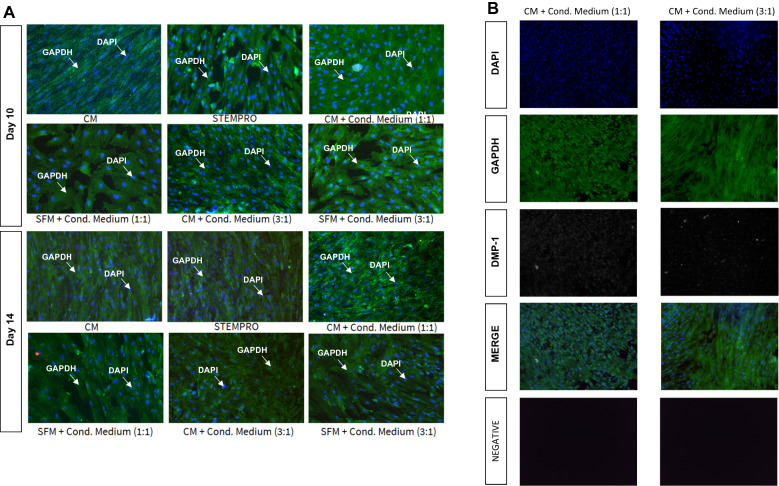
**A** Immunocytochemistry fluorescence images showing Day 10 and Day 14 induction on WJMSCs with the treatment aforementioned. **B** Immunocytochemistry fluorescence images on different channel (DAPI, GAPDH, DMP-1) on Day 14 induction. All fluorescence images were obtained at 10 × magnification. Immunocytochemistry utilizing GAPDH (green), DAPI (blue), and DMP-1 (red) dyes. *CM* complete media, *Cond. Medium* conditioned media, *SFM* serum free media

To detect characteristics of the odontoblast phenotype in induced WJMSCs, immunocytochemical staining for dentin matrix protein 1 (DMP1) was performed (Fig. 4A). The results indicated that the WJMSCs treated with complete media and DPSCs-CM with ratio 1:1 and 3:1for 14 days were minimally stained for DMP1, while no staining was observed in the control cells and the differentiated cells in the other treatments using the immunocytochemical assay (Fig. 4B).

## Discussion

In the present study, MSCs were successfully isolated from human Wharton’s Jelly. The isolated cells were in accordance to the minimal criteria to define human MSC as stated by ISCT [16]; the cells were shown to be plastic adherent, fibroblastic morphology, expressed positive markers such as CD90 (also known as Thy-1, a glycoprotein presented on the surface of MSCs, was related to the stemness of the cells [17]), CD105 (known as Endoglin, was responsible in angiogenesis process [18]), CD73 (glycoprotein that believed to be responsible in immune tolerance [19]) and lack expression of negative markers CD34, CD11b, CD19, CD45, HLA-DR (Fig. 1B). In this study, CD44, a hyaluronan receptor, which play a critical role in cell adhesion and migration, was also added in the positive markers panel to further characterise the population isolated cells. It was observed that the MSCs growth were significantly decreased after DPSC conditioned media induction, which indicating the transition of actively proliferation into differentiation state. The proliferative and differentiation potential of cells demonstrate an inverse relationship. Stem cells actively divide before maturing into a differentiated state, whereas terminal differentiation often occurs with and end to division cycle. The trade-off between continuation of cell-cycle and differentiation occurs with induction of cell type-specific genes and altered expression of cell cycle regulators.

DPSCs have been demonstrated to secrete factors that include cytokines and growth factors. Murakami et al. showed that DPSC-conditioned medium promotes odontoblastic differentiation of DPSC in vitro [20]. This occurrence was attributed to the presence of high levels of cytokines in the DPSC secretome such as Neurotrophin 3 (NT3),Bone Morphogenetic Protein (BMP) and DMP-1 (Dentin Matrix Protein-1) which are involved in odontoblast differentiation [20, 21]. Expression of NT3 is upregulated and augments differentiation of dental MSC into odontoblasts, while DMP is also known to promote odontoblastic differentiation. In this study, DMP-1 was used to detect odontoblastic differentiation in Wharton’s Jelly MSCs. The low expression of DMP-1 shown in immunocytochemistry indicating that DPSC conditioned media could possibly induce odontoblastic differentiation in MSCs. However, the induction period could be extended to at least 21 days for better differentiation result [22–24].

Currently, the release of molecules and factors to extracellular environment is thought to be the cornerstone of the therapeutic potential of MSC. The secreted substances have paracrine activity and thus named secretome. This secretome includes proteins, nucleic acids, lipids and also extracellular vesicles. Thus far, as compared to our current method, DPSC secretome is collected via various methods depending on the study: it can be obtained from 24–72 h after cell conditioning and at different percentages of confluence (50–80%) or culture passages (passages 3–10) [25, 26]. In all of the previous studies, the authors starved DPSC or SHED by retaining culture medium with added serum or switching to a serum-free medium, or a low serum concentration [27]. Some authors centrifuged and some concentrated the collected culture medium and stored the supernatant with or without proteinase inhibitors [28]. The disparity in protocols for isolation and production of secretome complicate studies and therefore need to be standardized and optimized. The content of the secretome can be study thoroughly in order to obtain the best secretion factors which induce the differentiation effectively.

## Conclusion

In this investigation, DMP-1 was minimally expressed on day 14 which shows some odontoblast-like cells have differentiated from the WJMSC. DPSCs conditioned medium appeared as a potential odontoblastic-induction approach for WJMSCs by inducing formation of odontoblast-like morphology without significantly inhibiting the cell proliferation. To further investigate the stimulatory effects by DPSCs conditioned medium, specific signaling pathways need to be elucidated to enhance the differentiation efficiency.

## Methods

### Cell isolation and culture

To isolate DPSC, healthy permanent teeth were collected from adult donors (under orthodontic treatment; 18–25 years old) at the Faculty of Dentistry, Universiti Kebangsaan Malaysia, Kuala Lumpur. Umbilical cord has been collected at the Universiti Kebangsaan Malaysia Medical Centre with informed consent from donors who deliver full term by elective caesarean section. The protocol of this study had been approved by Research Ethics Committee Faculty of Medicine Universiti Kebangsaan Malaysia (FF-2019-444).

Briefly, Tooth was cleansed with povidone-iodine and split at the cemento-enamel junction using sterilized dental burs to extract the dental pulp. Dental pulp tissue then minced into smaller fragments with sterile blade and digested using 4 mg/mL collagenase Type I enzyme (Gibco, Grand Island, NY, USA) for 40 min at 37 °C in water bath. The tissue fragments were resuspended in complete medium containing α-MEM supplemented with 10% fetal bovine serum (FBS), 1% GlutaMAX (Gibco, Grand Island, NY, USA) and 1% antibiotic–antimycotic. The cells were cultured in humidified incubator with 5% CO_2_ at 37 °C with medium change every 3 days until the cells reaches 80% confluence.

The umbilical cord was washed with Dulbecco’s phosphate-buffered saline and stripped off the umbilical arteries and vein. Wharton’s jelly was then minced into 2mm^2^ size and digested with 0.6% collagenase type II at 37 °C under gentle agitation. Resulting tissue fragments were suspended in DMEM low glucose supplemented with 1% GlutaMax™ (Gibco), 1% Antibiotic–Antimycotic (Gibco), with addition of 10% FBS, 10% hPL, or 10% human serum. The cells were cultured in humidified incubator with 5% CO_2_ at 37 °C with medium change every 3 days until the cells reaches 80% confluence and harvested using 1X TryPLE Select. The cells were expanded to passage 3 for the following experiments in this study.

### DPSC conditioned medium collection

DPSCs were seeded at a density of 5000 cells/cm^2^ in culture flasks and grown until approximately 70–80% confluent. Cells then washed three times with PBS and incubated with freshly added serum-free DMEM containing penicillin–streptomycin at 24, 48 and 72 h, at 37 °C in 5% CO_2_. Supernatant then collected, centrifuged at 4 °C at 3000*g* for 3 min followed by 5 min at 1500*g*, filtered through 0.2-μm filters and stored in aliquots at − 80 °C as DPSC-CM.

### Analysis of cell viability

To identify MSC viability when inducing differentiation with DPSC-CM, 3-(4,5-dimethylthiazoiyl-2)-2,5-diphenyltetrazolium bromide (MTT) coloration has been done. MSC were seeded at a density of 5000 cells/cm2 with the following media ratio: (a) Complete DMEM:CM 1:1; (b) Complete DMEM:CM 3:1; (c) Serum Free DMEM:CM 1:1; (d) Serum Free DMEM:CM 3:1. The cells were cultured in humidified incubator with 5% CO_2_ at 37 °C for 7 days with medium change every 3 days. MTT assay was done on day 1, 3, 5, and 7 to evaluate the effects of DPSC-CM towards MSC viability.

### Differentiation of MSC into odontoblast

Four complete αMEM:CM combination aforementioned were used for MSC odontoblast differentiation induction, whereas StemPro™ Osteogenesis induction media (Gibco) was served as positive control treatment. The cells were differentiated for 7 and 14 days prior subsequent experiment.

### Alizarin Red staining

At the end of each treatment, differentiated cells described above were stained with Alizarin Red to show positive calcium deposition. The staining dye was first observe under inverted microscope and then quantified using a mixture of 20% methanol and 10% acetic acid in distilled water for elution, the eluted dye was analyse using spectrophotometer at wavelength 450 nm.

### Immunocytochemistry detection of odontogenic marker

Expression level of DMP-1 was evaluated using immunocytochemical analysis protocol reported by [2] with slight modification. In brief, cells were washed with DPBS and fixed with 4% paraformaldehyde (PFA) for 30 min, followed by cell permeabilization by 0.5% Triton X-100 solution for 20 min, and then blocked with 5% Bovine Serum Albumin for 1 h at 37 °C. 1:200 mouse anti DMP-1 antibody and 1:200 rabbit anti-GAPDH were incubated with the cells overnight. On the following day, the cells were washed before being incubated with 1:300 diluted Alexa Fluor 594 anti-rabbit IgG and Alexa Fluor 488 anti-mouse for 1 h at 37 °C. Nuclei were counterstained with DAPI. Fluorescence images were captured with a Nikon Eclipes Ti Fluorecence microscope.

### Statistical analysis

Experiments were performed in triplicate and repeated on at least three biological samples (n = 3), and data are presented as mean ± SD. For statistical analysis, ANOVA was used. Statistical analysis was performed using Prism Version 7.0 software. Results were considered statistically significant at *P* < 0.05. All values are expressed as mean ± SD.

## Data Availability

The datasets used and/or analysed during the current study are available from the corresponding author on reasonable request.

## Abbreviations

DPSC: Dental pulp stem cell
WJMSC: Wharton’s jelly mesenchymal stem cells
MTT: 3-(4,5-Dimethylthiazol-2-yl)-2,5-diphenyltetrazolium bromide
ICC: Immunocytochemistry
DMP-1: Dentin matrix acidic phosphoprotein 1
ARS: Alizarin Red staining
PMMA: Polymethylmethacrylate
DPSCs-CM: Dental pulp stem cell conditioned media
NT3: Neurotrophin 3
BMP: Bone morphogenic protein
